# 

**Published:** 1894-09

**Authors:** 


### CONTENTS:

### SELECTIONS.

Article I.—Local Obtundents. By A. W. Haidle, D. D. S. - 193
" II.—Right-Sightedness and Left-Sightedness. By M. De Camarasa. - - - - - - - - 213
" III.—National Association of Dental Faculties. - - 216
" IV.—The Treatment of Typhoid Fever. By Elmer Lee, A. M., M. D. - - - - - - - 223
" V.—Sodium Peroxid Solution. - - - - 229
" VI.—Caries of the Alveolus. - - - - - - 230

### COMMUNICATED.

American Dental Society of Europe. - - - - - - 233

### MONTHLY SUMMARY.

A Case in Practice. - - - - - - - - 234

Hot Water for Hemorrhage. - - - - - - 235

Amblyopia Caused by Dental Irritation. - - - - 236

Bleaching With Pyrozone. - - - - - - 236

Cleaning the Teeth. - - - - - - - - 236

The Use of Peroxide of Hydrogen as a Hæmostatic. - - - 237

Keeping the Gingival Portion of a Cavity Dry. - - - 238

Gold in Pauper's Teeth Saved. - - - - - - 239

To Secure Rubber Dam. - - - - - - - 240

Treatment of Epithelioma. - - - - - - 240

